# MiR-29b controls fetal mouse neurogenesis by regulating ICAT-mediated Wnt/*β*-catenin signaling

**DOI:** 10.1038/cddis.2014.439

**Published:** 2014-10-16

**Authors:** J Shin, Y Shin, S-M Oh, H Yang, W-J Yu, J-P Lee, S-O Huh, S H Lee, Y-H Suh, S Chung, H-S Kim

**Affiliations:** 1 Department of Pharmacology and Biomedical Sciences, Seoul National University, College of Medicine, 103 Daehakro, Jongro-gu, Seoul 110-799, South Korea; 2 School of Mechanical Engineering, Korea University, Seoul 136-713, South Korea; 3 Department of Pharmacology, College of Medicine, Hallym University, Chuncheon 200-702, South Korea; 4 Department of Physiology and Biomedical Sciences, Seoul National University, College of Medicine, 103 Daehakro, Jongro-gu, Seoul 110-799, South Korea; 5 Center for Stem Cell Research and Regenerative Medicine, Department of Pharmacology, Tulane University School of Medicine, New Orleans, LA 70112, USA; 6 Department of Neurosurgery, Boramae Hospital, Seoul National University, Dongjakku, Seoul 156-707, South Korea; 7 Korea Brain Research Institute (KBRI), Daegu 700-010, South Korea; 8 Seoul National University Bundang Hospital, Seoul National University, College of Medicine, Sungnam 463-707, South Korea

## Abstract

*β*-Catenin has been widely implicated in the regulation of mammalian development and cellular homeostasis. However, the mechanisms by which Wnt/*β*-catenin signaling components regulate physiological events during brain development remain undetermined. Inactivation of glycogen synthase kinase (GSK)-3*β* leads to *β*-catenin accumulation in the nucleus, where it couples with T-cell factor (TCF), an association that is disrupted by ICAT (inhibitor of *β*-catenin and T cell factor). In this study, we sought to determine whether regulation of ICAT by members of the microRNA-29 family plays a role during neurogenesis and whether deregulation of ICAT results in defective neurogenesis due to impaired *β*-catenin-mediated signaling. We found that miR-29b, but not miR-29a or 29c, is significantly upregulated in three-dimensionally cultured neural stem cells (NSCs), whereas ICAT is reduced as aged. Treatment with a miR-29b reduced the reporter activity of a luciferase-ICAT 3′-UTR construct whereas a control (scrambled) miRNA oligonucleotide did not, indicating that miR-29b directly targets the 3′-UTR of ICAT. We also found that treatment with miR-29b diminished NSC self-renewal and proliferation, and controlled their fate, directing their differentiation along certain cell lineages. Furthermore, our *in vivo* results showed that inhibition of miR-29b by *in utero* electroporation induced a profound defect in corticogenesis during mouse development. Taken together, our results demonstrate that miR-29b plays a pivotal role in fetal mouse neurogenesis by regulating ICAT-mediated Wnt/*β*-catenin signaling.

Neural stem cells (NSCs), which originate in the developing mammalian brain, are defined by their specialized capacity to self-renew, proliferate, and differentiate into multiple cell lineages.^1^ As a result of their multipotency, NSCs can give rise to neurons, astrocytes, and oligodendrocytes.^2^ NSCs in the developing brain are dependent on signals from neighboring cells and extracellular matrix (ECM) proteins. Experimentally, this surrounding environment can be mimicked using a 3-dimensional (3-D) culture assay that enables microscale cell–cell and/or cell–ECM interactions and quantitative investigation of NSC self-renewal and differentiation.^3,4^

Wnt/*β*-catenin signaling has been extensively implicated in embryonic development and tumorigenesis.^5,6^ *β*-Catenin, a key component of the Wnt signaling pathway, translocates to the nucleus and binds to members of the lymphoid enhancer binding factor (LEF)/T-cell transcription factor (TCF) family of transcription factors^7^ to initiate expression of Wnt-responsive genes such as *cyclin D1*^8^ and *c-myc*. ICAT (inhibitor of *β*-catenin and TCF), also known as CTNNBIP1, directly interacts with *β*-catenin via three helical domains.^9^ This binding prevents the interaction of *β*-catenin with TCF and interferes with Wnt signaling mediated by the *β*-catenin–TCF complex.^10^ ICAT-knockout mice develop severe forebrain malformation beginning at embryonic day E11.5.^11^ ICAT-deficient mouse embryos exhibit a smaller overall brain size and abnormal forebrain morphologies. Therefore, the negative regulation of Wnt signaling by ICAT via competition with TCF for *β*-catenin is critical for brain development, most likely through promotion of NSC proliferation and differentiation.

MicroRNAs (miRNAs) are noncoding small RNAs 22 to 24 nucleotides in length. Endogenous miRNAs can silence target mRNAs by triggering endonuclease cleavage or promoting translational repression. The miRNAs that are expressed in NSCs can influence neurogenesis by fine-tuning gene expression.^12^ Previous studies have addressed whether the miRNA expression pattern in the brain drastically changes during embryonic development by screening the miRNA profiles of embryonic and adult forebrain, including cortex and striatum. These studies have shown that expression of the miR-29 family is absent in the embryonic brain, but is upregulated in the adult cortex and striatum.^13^ Using the bioinformatics programs, Targetscan (http://www.targetscan.org)^14^ and microRNA.org (http://www.microrna.org),^15^ we predicted potential molecular targets of miR-29b; among the predicted target molecules was ICAT. In the meantime, in addition to miR-29 family, many miRNAs including miR-328, miR-204, and miR-211 have been predicted to target ICAT by using the bioinformatics programs.

Here, we sought to determine whether regulation of ICAT by members of the microRNA-29 family is involved in neural development during early embryogenesis, and investigated the underlying mechanism. To reconstitute an *in vivo*-like microenvironment, we 3-D cultured NSCs in type I collagen ECM hydrogel incorporated in a microfluidic assay. Our results suggest that miR-29b, a member of the brain-enriched miR-29 family, acts through regulation of ICAT-mediated Wnt/*β*-catenin signaling to play a role in fetal mouse neurogenesis.

## Results

### MiR-29b is significantly upregulated during differentiation in NSCs

**Figure 1:**
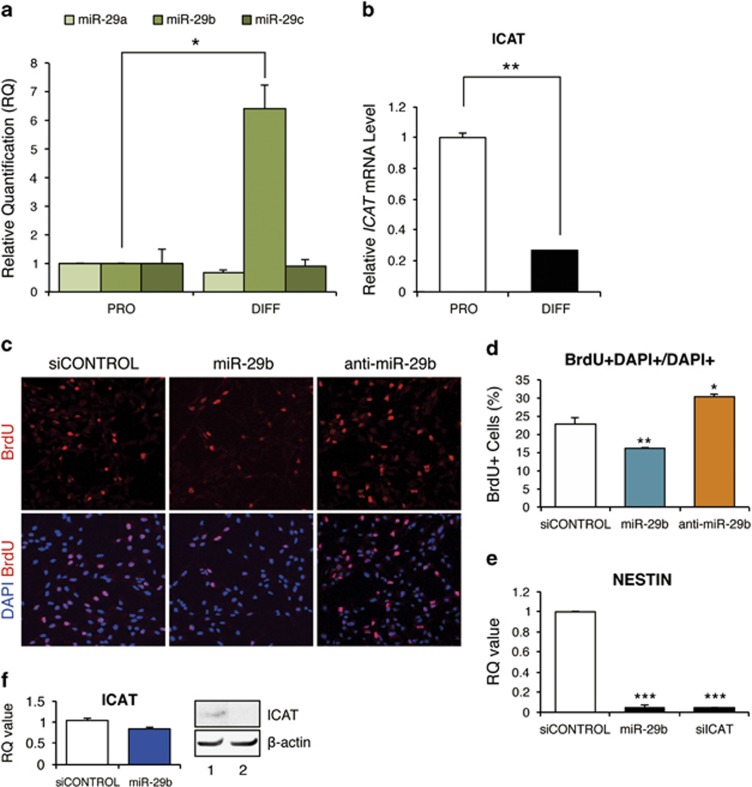
MiR-29b reduces NSC proliferation. (**a** and **b**) NSCs were harvested and the expression of miR-29 subtypes a, b, and c was quantified by qRT-PCR. MiR-29b, but not miR-29a or c, showed a prominent (approximately sixfold) increase following induction of differentiation. ICAT was highly expressed in fetal NSCs, but its expression declined with differentiation. (**c** and **d**) Fetal NSCs transfected with miR-29b or anti-miR-29b were plated on laminin-coated dishes, cultured in medium supplemented with EGF and FGF2 for 24 h, and labeled with a 1-h pulse of BrdU. Cells exhibiting a BrdU signal in DAPI-stained nuclei were considered positive. Approximately 1000 cells were quantified for each section. (**e**) Fetal NSCs transfected with miR-29b or siICAT were analyzed by qRT-PCR. Nestin expression was reduced by ~90% in ICAT-deficient NSCs. (**f**) Fetal NSCs transfected with miR-29b were analyzed by qRT-PCR and western blotting (1, siCONTROL; 2, miR-29b). The miR-29b transfection remarkably inhibited ICAT protein expression but ICAT mRNA was reduced by only ∼15% in NSCs (*P*=0.0525, Student's *t*-test, **f**). Data are expressed as mean±S.E.M. (error bars) of triplicate experiments (*P<0.05, **P<0.01, ***P<0.005; Student's *t*-test)

We performed quantitative real-time reverse transcription-PCR (qRT-PCR) analyses of miR-29a, b, and c subtypes to investigate the role of the miR-29 family in NSCs. MiR-29b, but not miR-29a or c, showed a prominent (approximately sixfold) increase following induction of differentiation using commercially available supplements (Stem Cell Technologies, Vancouver, BC, Canada) (*P*<0.05, Student's *t*-test, Figure 1a). A qRT-PCR analysis showed that ICAT mRNA levels were decreased by ∼80% in NSCs under differentiation media compared with those grown under proliferation conditions supplemented with epidermal growth factor (EGF) and fibroblast growth factor 2 (FGF2) (*P*<0.01, Student's *t*-test, Figure 1b).

### MiR-29b reduces NSC proliferation and self-renewal

To investigate the effect of miR-29b on NSCs, we grew E13.5 cortical cells that had been transfected with miR-29b or antisense 2′-*O*-methyl oligonucleotide against miR-29b (anti-miR-29b) as neurospheres and plated them in proliferation medium. A BrdU (5-bromo-2′-deoxyuridine) incorporation assay performed in proliferation medium revealed that BrdU-labeled DNA was decreased by ∼30% at 24 h in miR-29b-treated NSCs compared with scrambled miR-transfected cells (*P*<0.01, Student's *t*-test, Figure 1c), indicating a decrease in the propagating cell population. In contrast, anti-miR-29b promoted a 33% increase in BrdU-incorporating NSCs, compared with scrambled miR-transfected cells (*P*<0.05, Student's *t*-test, Figure 1c).

The EGF- and FGF2-responsive NSCs are present in the embryonic telencephalic germinal region as early as embryonic day 13.5 (E13.5).^16,17^ Early in development, most primitive neural progeny cells express the progenitor marker molecule, Nestin, a type VI intermediate filament protein. To examine the population ratio of self-renewed NSCs generated from neurosphere-expanded stem cells, we analyzed Nestin expression in miR-29b-transfected NSCs 3-D cultured in ECM by qRT-PCR. Nestin expression was significantly reduced in NSCs transfected with miR-29b or small interfering RNA (siRNA) against ICAT (*P*<0.005, Student's *t-*test, Figure 1e). In addition, we checked the effects of miR-29b transfection on the mRNA and protein levels of ICAT. Transfection of miR-29b remarkably inhibited ICAT protein expression but ICAT mRNA was reduced by only ∼15% in NSCs (*P*=0.0525, Student's *t*-test, Figure 1f). Figure 1b shows that miR-29b was significantly upregulated in 3-D NSC cultures and that the ICAT mRNA level was reduced in the differentiation medium by ∼80% (*P*<0.01, Student's *t*-test). The discrepancies in the statistical significance and reduction ratio between the results shown in Figures 1b and f may be explained by differences in the transfection efficiency of an exogenous miR-29b RNA oligonucleotides in primary NSC cultures.

### Nuclear *β*-catenin is necessary for differentiation of 3-D cultured NSCs

**Figure 2:**
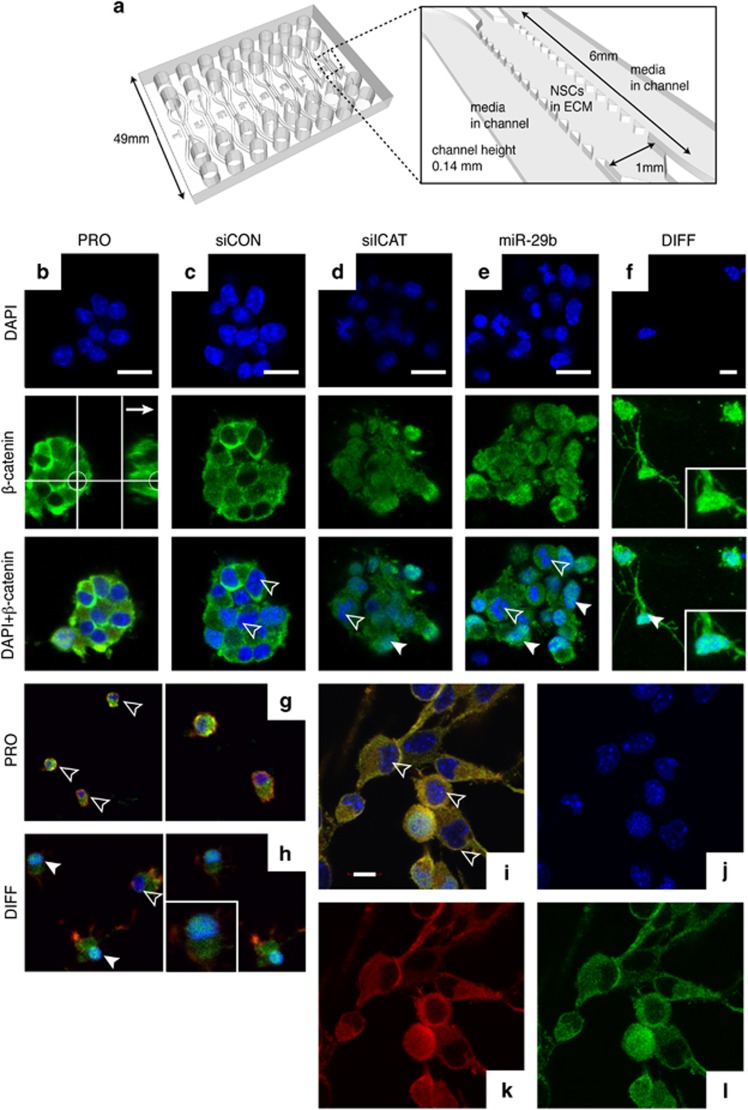
Nuclear *β*-catenin is necessary for NSC differentiation in 3-D culture. (**a**) Schematic of microfluidic assay for 3-D NSC culture in ECM. (**b**–**h**) *β*-Catenin was enriched in the nuclei of differentiated NSCs. Scale bars=15 *μ*m. Microscopy was performed on a Zeiss LSM 700 confocal laser-scanning microscope (Carl Zeiss, Jena, Germany) or an Olympus FV1000-MPE confocal/multiphoton microscope (Olympus, Tokyo, Japan). The 3-D reconstruction was performed using Imaris (custom software developed by Bitplane Scientific Software, Zurich, Switzerland). (**i**–**l**) ICAT colocalized with *β*-catenin in the cytosol in the presence of the growth factors such as FGF2 and EGF

*β*-Catenin translocates to the nucleus where it binds to members of the LEF/TCF family of transcription factors to induce expression of their target genes.^7^ It has not been clear where ICAT functions, although it has been shown that ICAT competes with TCF4, leading to inhibition of *β*-catenin–TCF4 complex.^5^ We found that transfection of 3-D NSCs in ECM with miR-29b or siRNA targeting ICAT (siICAT) triggered nuclear translocation of a *β*-catenin (Figures 2b–l), suggesting that this translocation event is essential for NSC differentiation (Figures 2b–f). We surmised that the *β*-catenin–ICAT complex is inactivated while in the cytoplasm, and that once released from ICAT, *β*-catenin becomes activated and subsequently translocates to the nucleus to initiate Wnt-responsive gene transcription. Overall, little has been known about the mechanisms controlling cortical development and the genetic pathways that regulate them. Our results provide evidence that ICAT cooperates closely with *β*-catenin in developing cortical layers. Therefore, ICAT depletion in NSCs in ECM by transfection with miR-29b or siICAT predominantly promotes differentiation toward intermediate progenitor cells via nuclear *β*-catenin.

### MiR-29b directly targets the 3′-UTR of ICAT

**Figure 3:**
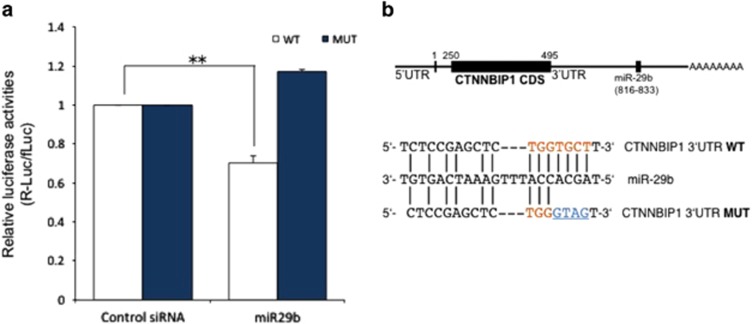
MiR-29b directly targets the 3′-UTR of ICAT. (**a**) HEK293T cells were transfected with luciferase reporter constructs containing WT or MUT (TGCT to GTAG) 3′-UTR of ICAT. Co-transfection of miR-29b with these reporters resulted in a decrease in the luciferase reporter activity of the WT 3′-UTR, but not the MUT 3′-UTR, indicating that miR-29b directly binds to the ICAT 3′-UTR. Firefly reporter luciferase activity was measured and normalized to *Renilla* activity (**P<0.005, Student's *t*-test). (**b**) Nucleotide pairing of miR-29b and *CTNNBIP1* 3′-UTR. The conserved miR-29b binding site is indicated by red letters; mutated bases are shown in blue

The TargetScan^14^ and microRNA.org^15^ algorithms showed that miR-29b targets the 3′-UTR of ICAT. The miR-29 family members were predicted to target a conserved sequence (TGGTGCT) in the 3′-UTR of ICAT. To validate whether miR-29b targets ICAT through this putative miR-29b binding site in the 3′-UTR, we used a luciferase reporter construct in which the mouse ICAT 3′-UTR (2173 bp) containing the predicted miR-29b binding sequence is positioned immediately downstream of the luciferase gene. The miR-29b oligonucleotides were co-transfected into human embryonic kidney HEK293T cells with wild-type (WT) or mutant (MUT) reporter constructs. Luciferase reporter activity of WT constructs was significantly decreased (~25%) in cells co-transfected with miR-29b (*P*<0.01, Student's *t*-test, Figure 3a). To determine whether the predicted miR-29b target site in the ICAT 3′-UTR is required for repression of ICAT expression, we mutated the TGCT sequence located within the conserved binding site to GTAG (Figure 3b), thereby abolishing miR-29b binding to the MUT construct. The activity of the reporter gene containing the MUT 3′-UTR was not affected by miR-29b (Figure 3a), indicating that miR-29b represses ICAT protein expression by specifically binding to the predicted target sites in the ICAT 3′-UTR.

### Inhibition of miR-29b induces a profound defect in corticogenesis during brain development

**Figure 4:**
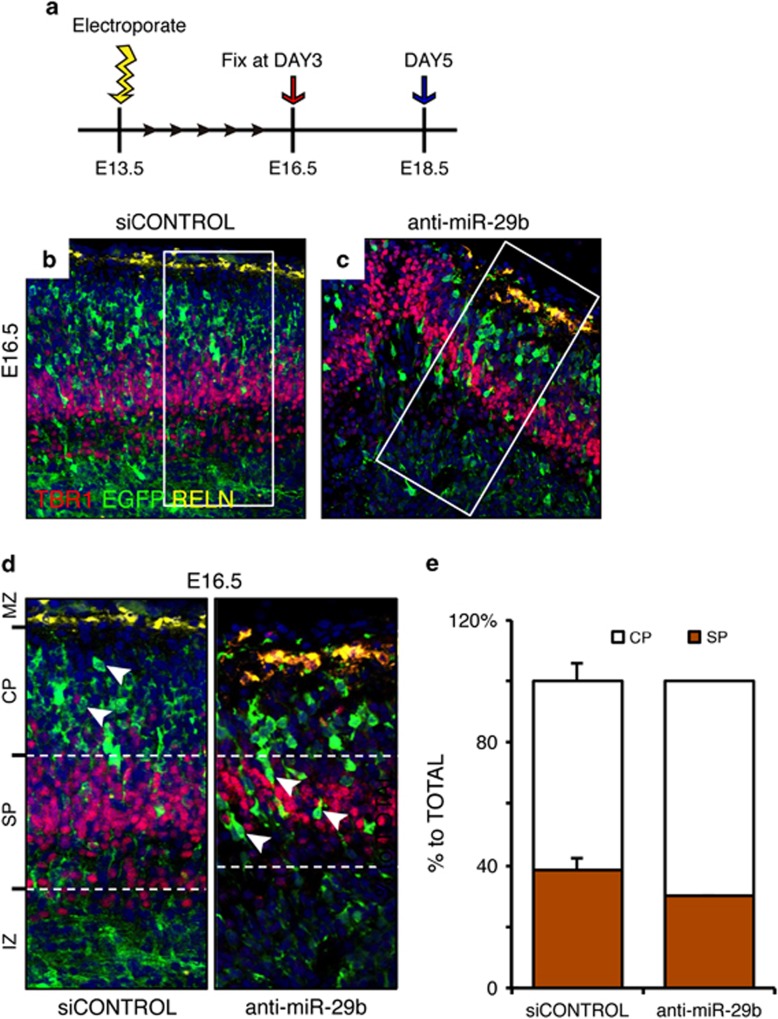
Inhibition of miR-29b induces a profound defect in corticogenesis during brain development. (**a**) Experimental scheme for *in utero* electroporation. RNA duplexes were electroporated at E13.5, and mouse cerebral cortices were harvested at E16.5 and E18.5. (**b**–**e**) The cortical plate at E16.5 shows TBR1-positive early pyramidal neuronal cells (red) and reelin (RELN)-expressing cells in the marginal zone (yellow). Anti-miR-29b oligonucleotides were microinjected into the left ventricle of mouse embryos at E13.5. Inhibition of miR-29b by *in utero* electroporation of anti-miR-29b into embryonic mouse brains led to premature outward cortical migration. Cells transfected with control siRNA radially migrated at the cortical marginal zone and showed a normal distribution of reelin expression. In contrast, reelin-positive Cajal–Retzius neurons in the marginal zone were decreased in brains microinjected with anti-miR-29b. MZ, marginal zone; CP, cortical plate; SC, subplate cells; IZ, intermediate zone, SVZ, subventricular zone; VZ, ventricular zone. Data are expressed as mean±S.E.M. values (error bars) or assays performed in triplicate

To determine whether our *in vitro* results could be substantiated *in vivo*, we microinjected an anti-miR-29b oligonucleotide into the left ventricle of mouse embryos at E13.5 (Figure 4a). Inhibition of miR-29b by *in utero* electroporation of anti-miR-29b into embryonic mouse brains led to premature outward cortical migration (Figures 4b–e). In contrast, knockdown of ICAT by RNAi-mediated gene silencing *in utero* resulted in proper cortical migration (data not shown). Cells transfected with control siRNA radially migrated at the cortical marginal zone and showed a normal distribution of reelin expression. In contrast, a decrease in reelin-positive Cajal–Retzius neurons in the marginal zone was observed in brains microinjected with anti-miR-29b, presumably because TBR-1 (T box brain gene-1) expression in the cortical plate is suppressed in the presence of anti-miR29b. TBR-1 is an early pyramidal neuron marker that is expressed toward the pial surface and in the cortical plate.^18^ Furthermore, GFP-positive premature neuroblasts showed excessive migration toward upper cortical plate layers compared with control siRNA-treated neuroblasts.

## Discussion

*β*-Catenin is a well-known protein involved in the regulation of vertebrate brain development. However, the mechanisms by which Wnt/*β*-catenin signaling components such as ICAT regulate biological events in the CNS remain undetermined. This research aimed to determine whether the miR-29 families regulate the expression of ICAT and, if so, whether deregulation of ICAT by miR-29 results in defective neurogenesis and whether these pathologies are because of impaired *β*-catenin-mediated signaling events. Here we demonstrate that miR-29b regulates *β*-catenin–ICAT complex during fetal neurogenesis.

**Figure 5:**
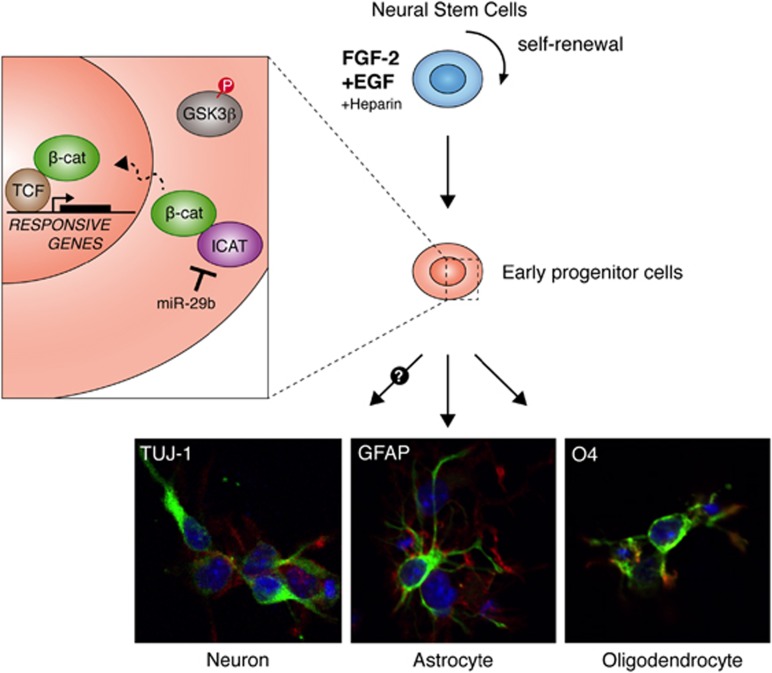
A schematic representation of Wnt/*β*-catenin-mediated neurogenesis in the CNS of prenatal mammals. A putative schematic model of ICAT-dependent neurogenesis showing that miR-29b targets ICAT, thereby facilitating proliferation of neural stem cells in the developing brain

The molecular aspects of the downstream components of *β*-catenin signaling have not been well characterized. Given that (1) miRNA-29 family members bind directly to ICAT, (2) *β*-catenin that is not bound to ICAT translocates to the nucleus, and (3) anti-miR-29b alters the functional properties of ICAT, it seems likely that ICAT is a component of *β*-catenin signal pathways (Figure 5). Although the genetic targets regulated by *β*-catenin and ICAT remain to be defined, our data provide a link between regulation of ICAT by miR-29b and corticogenesis. Furthermore, these results may contribute to a better understanding of the role of miR-29 miRNAs in the progression of many types of cancer, especially brain cancer. The miRNA molecules might also be considered as putative therapeutics in human patients with malignant glioblastoma multiforme (GBM), the most common type of primary brain tumor.

NSCs and brain tumor stem-like cells share the characteristics of the morphological and functional similarities such as self-renewal, extensive proliferation, and invasiveness.^19^ These research findings may identify novel therapeutic strategies to treat or prevent neurological diseases such as malignant GBM in humans.

## Materials and Methods

### Preparation of primary neurospheres from embryonic mouse brains

Timed-pregnant mice (CD1 albino, ICR) were purchased from Harlan Inc. animal care facilities (Indianapolis, IN, USA). Experimental procedures were performed in accordance with protocols approved by the Seoul National University Institutional Animal Care and Use Committee (SNU-130531-1). Embryonic neurosphere cultures were established by harvesting mouse cerebral cortices on E13.5, and dissociating and resuspending cortical cells.^16^

### cDNA synthesis and qRT-PCR analysis

The expression pattern of mature miR-29a, b, and c was assayed by quantitative RT-PCR. Briefly, 100 ng of total RNA, including small RNAs >18 nt in length, were reverse transcribed using an miScript starter kit (Qiagen, Hilden, Germany) according to the manufacturer's protocol. Specific RT primers for the miR-29b sequence, guide strand (5′-UAGCACCAUUUGAAAUCAGUGUU-3′ Qiagen), or U6 RNA were used. The cDNA was amplified using an ABI StepOne system (Applied Biosystems, Foster City, CA, USA). Relative quantification was carried out using the ΔΔCT method. Sample variability was controlled for by normalizing to U6 RNA levels. The miR-29b-regulated target mRNAs, including ICAT mRNA, were investigated by reverse transcribing 0.5 *μ*g of total RNA into cDNA followed by PCR. The levels of target mRNA were normalized to those of glyceraldehyde-3-phosphate dehydrogenase (GAPDH).

### Microfluidic 3-D cell culture assay fabrication and preparation

The microfluidic assay consisted of eight units of micro-patterned polydimethylsiloxane (PDMS; Sylgard 184; Dow Chemical, Midland, MI, USA) channels fabricated using a conventional soft lithography procedure. The sterilized micro-patterned PDMS and a glass coverslip substrate were treated with oxygen plasma (Femto Science, Seoul, Korea) and bonded together, forming a closed microfluidic channel. A single unit is composed of one center channel with two side channels. The center channel is for 3-D NSC culture and side channels are for growth medium supplementation.

### 3-D NSC culture in the microfluidic cell culture assay

For 3-D NSC culture, collagen type I (rat tail; BD Biosciences, Franklin Lakes, NJ, USA) was used as the ECM material. Collagen type I solution was diluted to 2 mg/ml in 10 × phosphate-buffered saline (PBS; Thermo Scientific, Waltham, MA, USA) and distilled deionized water, and the pH of the gel solution was adjusted to pH 7.4 with 0.5 N NaOH. E13.5 neurospheres were dissociated and suspended in ice-cold collagen solution at a density of 5 × 10^6^ cells/ml. Cultivated NSCs in collagen solution were injected into the central channel and allowed to gel by incubating at 37°C for 30 min. For cell transfection, Lipofectamine RNAiMAX (Invitrogen, Carlsbad, CA, USA) was mixed with 50 nM miRNA (Qiagen) or 100 nM siRNAs targeting ICAT (siICAT, SASI_Mm01_00054132; Sigma-Aldrich, St. Louis, MO, USA) from mouse (NM_023465). After gelation, complete proliferation medium and individual transfectants were added to the microfluidic assay through both side channels and incubated overnight. The cells were washed and the medium was refreshed daily. Specific gene expression in NSCs was quantified by qRT-PCR after 3 days in culture.

### Western blot analysis

Protein (30–50 *μ*g) from NSCs lysed in RIPA buffer was loaded onto 4–12% Bis-Tris gels (Invitrogen) and transferred to PVDF membranes (Millipore, Billerica, MA, USA). Each membrane was incubated with appropriate primary antibodies (ICAT, 1 : 500, Santa Cruz Biotechnology, Santa Cruz, CA, USA; *β*-actin, 1 : 5000, Sigma-Aldrich). *β*-Actin was used as loading control. The membrane was then incubated with anti-rabbit or anti-mouse secondary antibodies conjugated to horse radish peroxidase (HRP) (1 : 700–2000, Invitrogen), and visualized using an enhanced chemiluminescent (ECL) substrate (G-Biosciences, St. Louis, MO, USA).

### Immunocytochemistry

NSCs cultured in the microfluidic assay were fixed in 4% (w/v) paraformaldehyde in PBS at room temperature for 15 min and then washed with PBS. The fixed cells were permeabilized by incubating with 0.1% Triton X-100 for 5 min, blocked by incubating with 4% (w/v) bovine serum albumin (BSA) in PBS for 1 h, and washed with PBS. After blocking, cells were incubated with mouse monoclonal anti-TUJ1 (1 : 100; Millipore), mouse monoclonal anti-O4 (1 : 100; Millipore), rabbit polyclonal anti-GFAP (1 : 100; Abcam, Cambridge, UK), rabbit polyclonal anti-ICAT (FL-81) (1 : 100; Santa Cruz Biotechnology), or mouse monoclonal anti-active *β*-catenin (clone 8E7, 1 : 100; Millipore) antibodies for 2 h at room temperature. Cell nuclei were stained with 4',6-diamidino-2-phenylindole (DAPI; Sigma-Aldrich).

### MiRNA 3′-UTR target luciferase reporter assays

The full-length (2191 bp) 3′-UTR of the mouse ICAT (*CTNNBIP1*) gene on a pEZX-MT01 backbone (miTarget miRNA 3′-UTR Target Clones) was obtained from GeneCopoeia (Rockville, MD, USA). The MUT construct was created by substituting GTAG for the WT TGCT sequence within the miR-29-target binding site in the 3′-UTR. At 24 h after seeding, HEK293T cells were co-transfected with 0.5 *μ*g of reporter constructs (WT or MUT) and control siRNA or 50 nM mouse miR-29b miRNA oligonucleotides (Qiagen). After 24 h, luciferase activity was measured using the Dual-Luciferase Reporter Assay system (Promega, Madison, WI, USA) and normalized to *Renilla* luciferase activity.

### BrdU incorporation and immunostaining analysis

E13.5 NSCs were seeded at 1 × 10^5^ cells/well in laminin (Invitrogen)-coated 24-well plates. Cells were pulsed for 1 h with 10 *μ*M BrdU (BD Biosciences). The BrdU pulse-labeled cells were fixed and acid treated, followed by immunostaining with antibodies to BrdU (1 : 100; Abcam).

### *In utero* electroporation

Timed pregnant C57BL/6 N females at E13.5 were anesthetized with inhalational anesthetic, 2.5% isoflurane, and the uterine horns were exposed. Then, 1 *μ*l of small RNA (37.5 pmol of control siRNA or anti-miR-29b (5′-GCUGGUUUCAUAUGGUGGUUUA-3′)) together with 0.625 *μ*g of pCAGIG GFP-reporter plasmid DNA in PBS were microinjected into the lateral ventricle using 90-mm glass capillaries (GD-1; Narishige, Tokyo, Japan) as previously described.^20^ Electroporation was performed with Tweezertrodes electrodes (diameter, 5 mm; BTX, Holliston, MA, USA) using five 45-V pulses (50 ms duration, 950 ms interval) using a square-wave pulse generator (ECM 830; BTX).^21^ The embryos were allowed to continue growth, and analyzed at E16.5 and E18.5.

### Statistical analysis

Quantitative data were expressed as mean±S.E.M. for *n*>3. Significant differences between two groups were tested with unpaired Student's *t*-test using SPSS software (Chicago, IL, USA). A *P*-value of <0.05 was considered significant, and *P*-values for specific comparisons are indicated in figure legends.

## Abbreviations

anti-miR-29b: 2'-*O*-methyl oligonucleotide against miR-29b
BrdU: 5-bromo-2'-deoxyuridine
EGF: epidermal growth factor
FGF2: fibroblast growth factor 2
ICAT: inhibitor of *β*-catenin and T cell factor
LEF: lymphoid enhancer binding factor
miRNA: microRNA
MUT: mutant
NSC: neural stem cell
siRNA: small interfering RNA
TBR-1: T box brain gene-1
TCF: T-cell transcription factor
WT: wild type
